# Precision medicine and implications in medical education

**DOI:** 10.1097/MS9.0000000000000298

**Published:** 2023-03-27

**Authors:** Pratik Lamichhane, Anushka Agrawal

**Affiliations:** Maharajgunj Medical Campus, Institute of Medicine, Kathmandu, Nepal

**Keywords:** genomics, medical education, precision medicine

## Abstract

Precision medicine (PM) is a revolutionary approach that gathers and analyzes massive amounts of data on patient's history, lifestyle, genetic, and environmental factors to tailor the most effective treatment for the patient. The low utilization of PM in healthsector today can be tackled with the introduction of PM in medical education. In near future, medical education will observe gradual integration of PM in undergraduate and postgraduate curricula. The increased need of training of faculties, protection of patient's data and the use of advanced technologies are the likely ramifications of introduction of PM in medical education and healthcare.

Modern medicine evaluates almost every patient based on a ‘signs and symptoms’ approach and treats in line with the results of standard tests available today. This is the most usual way of treating patients globally and is known as evidence-based medicine (EBM). To define, EBM is the process of making healthcare decisions based on the best available medical evidence, clinical expertise, and patient values. This practice involves making recommendations to patients based on data gathered from high-quality, evidence-based studies. A clinician must have a robust grasp of literature review and statistics interpretation in order to employ EBM. The clinician must then analyze and use the information, as well as his or her clinical expertise acquired throughout practice. The evidence can help clinicians, but it can never replace human clinical skills in determining whether the evidence applies to a specific patient. However, not all evidence is created equal! The study that provided the medical evidence determines its relative strength and quality^1^. A pyramid is commonly used to symbolize the evidence hierarchy. (Fig. 1) The outcomes of randomized controlled trials lie at the core of evidence adopted in EBM. Even though the importance of clinical trials is irrefutable, results from the trials represent the classic example of average treatment effect. Randomized trials focusing on average treatment effects tend to hide heterogeneity in individual patient responses^2^.

**Figure 1:**
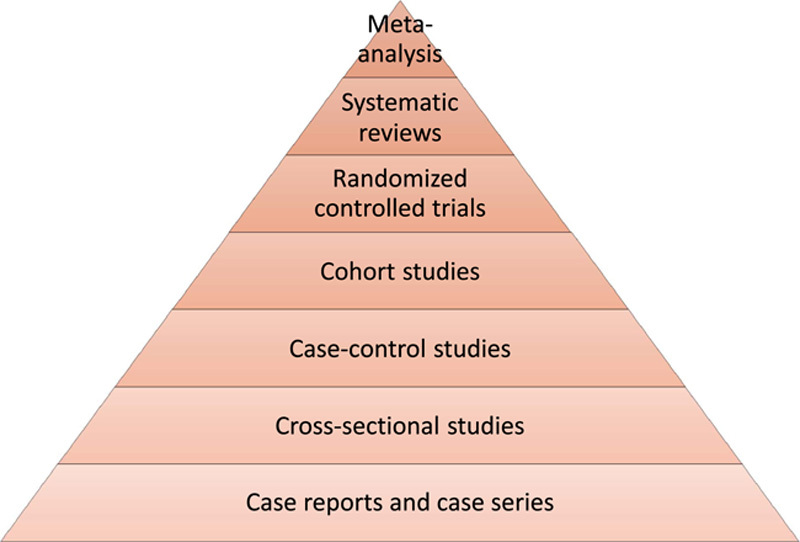
Hierarchy of studies in evidence-based medicine.

This method of treatment may be beneficial for most people, but not everyone observes a similar effect. These treatments are not patient-specific and are intended to treat a ‘typical’ or ‘average’ patient. This ends up leaving a portion of patients being cured or treated, whereas the remaining patients may not benefit at all. Specifically, a ‘one-size-fits-all’ strategy to treat cardiovascular illnesses, cancer, and diabetes may not always succeed. These diseases are the result of the complex interplay of genetic, environmental, and lifestyle factors. The trials conducted in most centers are unable to address heterogeneity among patients in terms of these factors. The selective enrollment of patients in a clinical trial according to the inclusion criteria frequently results in restricted generalizability of the outcomes generated by that study. This can lead to ineffective treatment of outlier patients who do not belong to the same patient subgroup that was included in the clinical study. This could be one of the reasons behind the inapplicability and failure of treatment among a certain proportion of patients for a particular disease^3^.

Even though EBM has been the most effective method for arriving at a diagnosis and prescribing a treatment to date, its limitations cannot be unseen. Randomized trials with positive results are more likely to be published than studies with neutral or negative results. This leads to a publication bias since systematic reviews and meta-analyses’ findings are based solely on published articles. Therefore, EBM may be insufficient in providing information on outlier cases^4^. Precision medicine (PM), which uses more detailed information to determine the most effective treatment option to manage the disease for every patient including outliers and nonresponders to the therapy administered in the trial, is one answer to this problem. PM is a revolutionary clinical approach that gathers and analyzes massive amounts of data on history, lifestyle, genetic, and environmental factors to tailor the most effective treatment for the patient^3^,^5^. The goals of PM are vast, holistic, and revolutionary. They consist of improved disease categorization, detection, early recognition of subclinical illnesses, monitoring of disease evolution, and improved management of the disease. The overall aim of PM is to provide the right kind of treatment to the right patient at the right time. Therefore, successful implementation of the PM can improve health outcomes and overall patient satisfaction^3^.

Let us consider a traditional scenario for an elderly male patient diagnosed with lung cancer. He visits an oncologist to receive the appropriate treatment. The oncologist enrolls him on a regimen of chemotherapy based on the diagnosis made from clinical findings and results of a biopsy, along with standard lab tests. But now, let us consider the same patient who instead visits a hospital in which PM is being practiced. The doctor orders a whole-genome sequencing test to identify specific mutations present in the patient’s DNA that are responsible for cancer. Furthermore, the doctor studied the patient’s lifestyle and environment to gain more insight into risk factors such as smoking and indoor and outdoor air pollution. Subsequently, he is classified into a subgroup of patients with the same mutations as his. He receives a proven targeted therapy specific to patients of his subgroup. The treatment approach in the second scenario is a simple example of a PM that provides uniquely responsive treatment and a better prognosis (Fig. 2).

**Figure 2:**
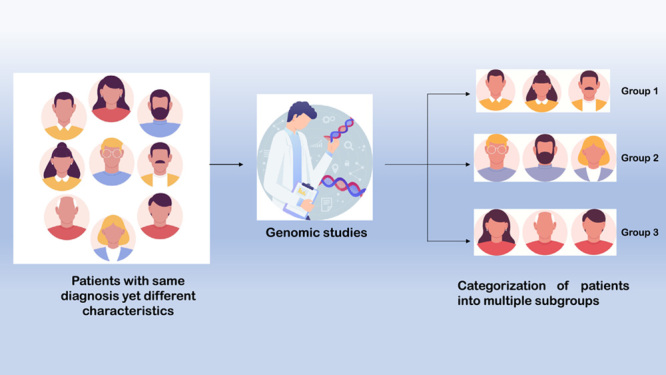
Outline of process of treatment in precision medicine.

Despite the differences between EBM and PM, it is necessary to bridge them in modern-day practice. We are currently at the phase of transition where reconciliation of EBM and PM is inevitable. PM offers a wide range of implications for patients, healthcare providers, and medical students. A new taxonomy of diseases, new genetic testing, and newer medicines will become available over time. Since molecular processes, demographics, environmental factors, general lifestyle, and dietary habits are researched in order to create a new patient identity, a large volume of patient data will be collected. This ‘big data’ includes molecular data on DNA (genomics), RNA (transcriptomics), proteins (proteomics), and metabolites (metabolomics)^6^. The collection of such massive data points and its utilization in daily practice is a challenging task. Millions of unique and complex data points can lead to a case being so unique that ‘N-of-one’ situations may be encountered. This creates difficulty in defining evidence-based guidelines in a PM since the statistical power gets very limited. This can be curbed with the coordination of multiple institutions with big data, which can be pooled together for meta-analyses. This aggregation of data from similar ‘N-of-one’ cases results into ‘N-of-many’ paradigms which allows us to draw reliable evidence for the stratified diseased population. Therefore, EBM and PM should be viewed as mutually complementary, and reconciliation provides us with an opportunity to merge the strengths of both approaches^3^.

Even though the PM is getting popular among health workers as well as the general public, adoption by clinicians into the practice is surprisingly low^7^. A common trend of hesitancy among physicians to apply advancements in medical science has been observed^8^. Such reluctance among service providers can be tackled only with the introduction of PM from the undergraduate level in medical school^9^. The International Consortium for Personalized Medicine has the vision to produce a new generation of informed, empowered, engaged, and responsible healthcare providers by 2030 with the help of major changes in curricula^10^. The emergence of PM will fundamentally revolutionize the teaching–learning process in medical schools. Hence, medical schools should broaden their curricula to include practical training for the applications of genomic studies in clinical medicine in order to close the era of the gap in medical education^11^.

In spite of living in an innovative era of digital health, only a small proportion of medical students feel that their education has made them competent in the practice of PM. Likewise, it has been found that the PM content is discussed in only 21% of the undergraduate curriculum^12^. The expected barriers to providing training are heavy student academic workload and lack of instructions regarding scientific research. One medical school in the USA has used a spiral learning model to introduce PM in undergraduate medical education. This simple teaching–learning model comprised five distinct sessions on scientific advancements in PM. This program was integrated with the existing curriculum using a spiral learning model, which included building on foundational concepts with progressively deeper content coverage. The spiral model is able to keep up with the constantly evolving PM since each activity builds on and extrapolates previously presented information to the students. Topics such as the use of stem cells and cancer PM, along with elective courses, were covered in 4 years of undergraduate education. This model was deemed useful by more than 80% of its recipients. Such a method was also found to inspire students to look out for opportunities to learn about PM^9^.

Faculties are at the core of teaching–learning process in all medical schools. In order to facilitate the teaching, interested faculties can be encouraged to integrate PM into their clinical practice, teaching, and research. Interested faculty members could be enrolled in workshops that focus on genomic medicine topics relevant to the current medical practice. Such workshops also provide an additional opportunity for collaboration among faculty to initiate research and add didactic methods in teaching PM. Similarly, other teaching methodologies, such as ‘flipped classrooms’ have been utilized in some settings to indulge students in distinct topics of PM, such as precision diagnostics. Flipped classrooms area type of blended learning method where students are introduced to content at home and practice working at school. This approach has been found effective since the number of surplus live classes at school can be reduced, and the academic workload does not increase significantly^13^.

Since personal ‘-omics’ data may become a regular component of medical records, it is critical that current and upcoming medical students receive training in the utilization of the so-called ‘big data.’. These data sets are handled and interpreted by the field of clinical bioinformatics. With the rise of PM, the importance of bioinformatics will become predominant. Bioinformatics is likely to allow a shift from the traditional organ-based paradigm to a more holistic and systemic assessment of health and disease. Additionally, there has been an increasing importance of the medical speciality and subspecialities of clinical bioinformatics. This necessitates a profound reshaping of medical curricula to train highly specialized experts responsible for the interpretation of data and for issuing reports that will serve as decision support for physicians^3^. Hence, the new educational approach should teach students how to choose molecular tests, interpret the results, integrate findings with clinical history, and deliver tailored treatment to patients^11^. Protection of such personal data and confidential sharing among clinicians and researchers at the same time are equally crucial^14^. Therefore, students and young doctors shall be introduced to contemporary regulations to protect patients’ privacy.

Newer technologies can be leveraged to assist with this drastic change in medical education. Students and young internists may need to be trained in the new electronic health recording system software, which now incorporates diverse patient-specific information. Likewise, students should be acquainted with artificial intelligence and machine-learning technology, which might play a great role in the interpretation of big data for PM in the future^15^. A radical overhaul seems essential in medical education to produce a health workforce capable of utilizing PM optimally to deliver effective healthcare.

## Ethical approval

Not applicable.

## Consent

Not applicable.

## Sources of funding

None of the authors has received specific funding for the work.

## Author contribution

Both authors were involved in drafting and reviewing the manuscript and have seen the final text. Both of them contributed equally to the development of the manuscript.

## Conflicts of interest disclosure

The authors have no conflicts of interest to declare regarding this article.

## Research registration unique identifying number (UIN)

1. Name of the registry: NA.
2. Unique identifying number or registration ID: NA.
3. Hyperlink to your specific registration (must be publicly accessible and will be checked): NA.

## Guarantor

Pratik Lamichhane.

## Provenance and peer review

Not commissioned, externally peer-reviewed.
